# The presence of iteroparous salmonid spawning grounds affects the stable isotope signatures of food web components in Swedish boreal streams

**DOI:** 10.1016/j.heliyon.2025.e42173

**Published:** 2025-01-23

**Authors:** Rahmat Naddafi, Magnus Enbom, Carin Magnhagen, Nazila Hajizadeh Koupayh, Daniel Holmqvist, Hans Lundqvist

**Affiliations:** a Swedish University of Agricultural Sciences, Department of Aquatic Resources, Uppsala, Sweden; b Swedish University of Agricultural Sciences, Department of Wildlife, Fish, and Environmental Studies, Umeå, Sweden; c Ume/Vindelälvens Fiskeråd, Lycksele ommun, Lycksele, Sweden

**Keywords:** Atlantic salmon, Sea trout, Periphyton, Consumers, Stable carbon and nitrogen isotopes, Trophic ecology, Bayesian mixing model

## Abstract

Marine-derived nutrients (MDN) translocated by anadromous fish can strongly be linked to the dynamics and structure of resident freshwater communities. Yet there is limited knowledge on the transport of marine nutrients by Atlantic salmon (*Salmo salar*) and sea trout (*Salmo trutta*) from the Baltic Sea and their incorporation into the trophic system of the boreal streams. Here, stable carbon (δ^13^C) and nitrogen (δ^15^N) isotopes were measured in food web components (periphyton, benthic macroinvertebrates, and a predatory fish (freshwater-resident brown trout) of four sites with and without salmonid spawning grounds. Two sites (MDN sites) had spawning Atlantic salmon and sea trout and two other sites (reference sites) had no spawning or mortality areas of these species. A generalized linear mixed model revealed that site type and food web component had a significant effect on δ^15^N and δ^13^C values. All food web components studied were enriched with ^15^N and ^13^C in MDN sites where the anadromous salmonids spawn and experience high overwinter mortality. The average δ^15^N and δ^13^C values were 3.3 ‰ and −23.3 ‰ in periphyton, 4.3 ‰ and −28.9 ‰ in benthic macroinvertebrates, and 7.8 ‰ and −25.7 ‰ in brown trout in MDN sites, respectively. In reference sites, the average δ^15^N and δ^13^C values were 1.8 ‰ and −32.7 ‰ in periphyton, 2.0 ‰ and −36.9 ‰ in benthic macroinvertebrates, and 6.5 ‰ and −29.9 ‰ in brown trout, respectively. Upstream migrating Atlantic salmon were more ^15^N enriched than migrating sea trout. Both Atlantic salmon and sea trout eggs had higher δ^15^N values and lower δ^13^C values than their muscles. A Bayesian mixing model revealed variations in the diet of brown trout in different streams. The results from this study show that stream food web components have different isotopic signatures depending on whether iteroparous salmonid spawning/mortality occurs or not.

## Highlights

• We examined δ ^15^N and δ^13^C values of organisms in sites with and without spawning salmonids.
• Anadromous salmonids had high amounts of Marine-derived nutrients (MDN) in their bodies.
• Site type and food web component had a strong effect on the δ^15^N and δ^13^C values.
• All organisms studied were enriched with ^15^N and ^13^C in MDN sites where salmonids breed and die.
• MDN transferred by iteroparous salmonids can be incorporated into stream communities.

## Graphical abstract

**Figure:**
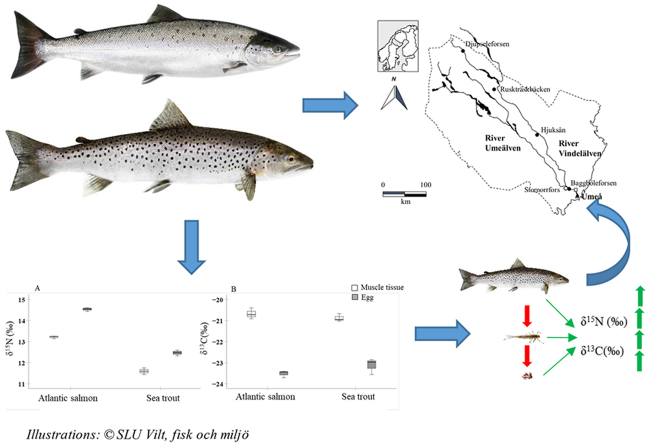

## 1 Introduction

Animals play an important role in nutrient cycling because they can either translocate nutrients across ecosystems or recycle nutrients within a habitat [1,2]. Especially, depending on the type of animal, nutrient translocation could occur across great spatial and temporal scales, stimulates “new primary production”, and increases the total amount of nutrients in the recipient ecosystem [2]. One of the best-studied examples of nutrient translocation is anadromous Pacific salmon (*Oncorhynchus* spp.) that are known to transport a large amount of nutrients hundreds of kilometers from the ocean to the North American freshwater ecosystems [[2], [3], [4], [5], [6]]. This species is born in freshwater, and after smoltification, they migrate to the ocean where they grow and accumulate the nutrients from the surrounding environment in their body. Most species of Pacific salmon have a semelparous life history, i.e., when they return to their natal streams as adult, they spawn once and then die. Thus, their spawning migration is associated with transferring a considerable amount of nutrients and energy into the freshwater ecosystem in the form of sperm, eggs, waste, and adult carcasses [2,5,7]. These nutrients are termed marine-derived nutrients (MDN) and are strongly linked to the dynamics and structure of resident freshwater communities [5,7,8]. Many studies have shown that these MDN can be incorporated into stream communities and surrounding riparian vegetation, stimulate primary production, and increase the biomass of macroinvertebrates and salmonid offspring [5,7,[9], [10], [11], [12], [13], [14], [15], [16]].

In contrast to Pacific salmon, anadromous European salmonids (Atlantic salmon *Salmo salar* and sea trout *Salmo trutta*) are iteroparous, such that adults that have survived the spawning may return to the ocean, leaving fewer carcasses in the river after spawning. In addition, these salmonids generally spawn at far lesser densities than the semelparous Pacific species, which would limit their MDN contribution. Thus, MDN present in Atlantic coast streams are likely limited to direct consumption of eggs by invertebrates and nutrient release from egg decomposition rather than through the incorporation of dissolved nutrients from carcass decay [17]. Available evidence from Norwegian waters indicates that 15–60 % of Atlantic salmon post-spawner mortality occurs in freshwater habitats [18]. Hence, less nutrients are transferred by anadromous European salmonids and retained in the freshwater habitat [19]. As a result, MDN has received less attention in Europe compared to the west coast of North America. Wild populations of Atlantic salmon have gone extinct in many Swedish rivers, and most of the present natural salmon production occurs in rivers connected to the northern part of the Baltic Sea. Similarly, overfishing is threatening sea trout stocks, potentially affecting the nutrient dynamics in Swedish streams. However, a few studies have shown that Atlantic salmon and/or migratory sea trout can be a major vector for transporting MDN to Atlantic rivers, which might be highly important for the productivity of these freshwater ecosystems [11,[15], [16], [17],[20], [21], [22], [23], [24], [25], [26], [27]]. Exposure to MDN resources from spawning Atlantic salmon has even enhanced the nutritional quality of all biota, as indicated by increased lipid stores and incorporation of fatty acids, contributing to the health of freshwater ecosystems [25]. Despite this potential importance, there has not been any use of stable isotope analyses to assess whether the presence of salmonid spawning grounds can change the isotopic signatures of food web components in the boreal streams through potential incorporation of MDN into the trophic system of these streams.

Stable isotope analysis provides a powerful tool to evaluate resource dynamics and trophic relationships in aquatic ecosystems [[28], [29], [30], [31], [32], [33]]. The ratio of heavy to light nitrogen isotopes (δ^15^N) is increased about 3.4 % from prey to predator and thus is used to quantify the trophic position of a consumer [34]. The ratio of heavy to light carbon isotopes (δ^13^C) is enriched little at each trophic level and hence is used to explore the origin and pathways of organic matter in food web [32,34,35]. Indeed, stable isotopic composition of food sources and trophic fractionation during the feeding process can determine isotopic values of the consumers. Since marine δ^15^N and δ^13^C are generally higher than freshwater δ^15^N and δ^13^C [32,36], stable isotope analysis can be used to trace the N and C from spawning salmonids through the trophic systems of streams they utilize [7]. For instance, the average δ^15^N and δ^13^C values of Age-1 cutthroat trout were 13.6 ‰ and −22.1 ‰, respectively, in an MDN stream compared to 8.1 ‰ and −27.5 ‰, respectively, in a non-MDN stream [7]. Nevertheless, isotopic enrichment does not necessarily mean importance given the complexities of isotope fractionation. It appears that most studies on isotopic values of large salmonids are on Pacific salmon [7,37] and Atlantic salmon [[38], [39], [40]]. However, no such studies have been found on large sea trout from the Baltic.

The main objective of this study was to evaluate whether the presence of salmonid spawning grounds has an effect on the stable isotope signatures of multiple stream food web components. Here, isotope ratios of N and C in Atlantic salmon (70–77 cm) and sea trout (57–69 cm) reproductive adults (females) were first analyzed to see if these migratory fish from the brackish Baltic Sea contained relatively high values of δ^15^N and δ^13^C in their bodies and eggs. Then, stable N and C isotope ratios in periphyton, benthic macroinvertebrates, and a predatory fish (freshwater-resident brown trout) collected from four similar-sized streams with and without salmonid spawning grounds were analyzed. The hypothesis was that food web components in streams supporting anadromous iteroparous salmonids have higher nitrogen and carbon isotope values compared to those in streams without salmonid spawning grounds. This study provides valuable insights into stable isotopic ratios of both large iteroparous Atlantic salmon and sea trout and the transfer of nutrients between marine and freshwater ecosystems.

## 2 Materials and methods

### 2.1 Study area and sampling

**Fig. 1:**
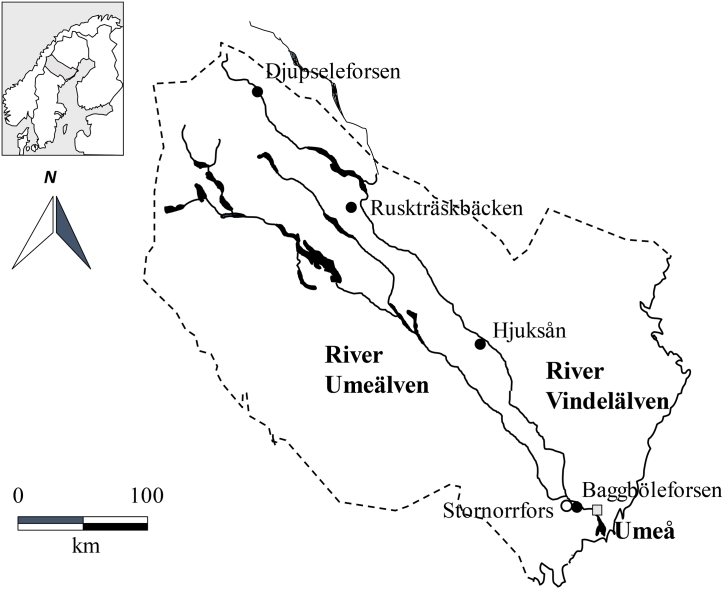
Study sites (bullet points) in the River Umeälven drainage area with its largest tributary River Vindelälven, Sweden. Marine-derived nutrients (MDN) sites (Baggböleforsen and Djupseleforsen; Reference sites (Hjuksån and Ruskträskbäcken). Hjuksån and Ruskträskbäcken are tributaries to Vindelälven, while Baggböleforsen and Djupseleforsen are located in the main part of Umeälven and Vindelälven. Fish were caught in the Stornorrfors fishladder (marked with open circle).

The rivers Umeälven and Vindelälven originate in parallel valleys with their headwaters in the mountains close to the Norwegian border, c. 450 km from the Bothnian Bay [41]. The Umeälven is dammed for hydroelectric power production throughout its length, so the passage of anadromous fish in this river is blocked by the first dam, Stornorrfors [41]. The Vindelälven merges with the Umeälven 12 km above Stornorrfors (64° N, 20° E) [41]. Anadromous Atlantic salmon and sea trout gain access to the Vindelälven by way of a fish ladder at Stornorrfors, located 32 km upstream from the coast [41]. This study was conducted in four sites (Baggböleforsen, Djupseleforsen, Hjuksån, Ruskträskbäcken) in the Umeälven drainage area with its largest tributary, Vindelälven (the county of Västerbotten, Sweden) (Fig. 1), with and without salmonid spawning grounds (see also supplementary materials for the details about streams; Table S1, Table S2). While Baggböleforsen is a stream with high winter mortality of salmon and sea trout downstream from the hydropower station in Stornorrfors, Djupseleforsen, with high densities of salmon and moderate densities of brown trout, is one of the largest spawning sites for salmon in the catchment area of the Vindelälven. Both Hjuksån and Ruskträskbäcken are medium sized tributaries to the River Vindelälven (Fig. 1) and have moderate to high densities of resident brown trout and no recorded spawning or catches of salmon. Mayflies, stoneflies, and caddisflies are likely the dominant benthic invertebrates in the sampling sites (see supplementary material, Table S1).

Hence, two sites [(Baggböleforsen (Umeälven) and Djupseleforsen (Vindelälven)] are considered as “MDN sites” where spawning and winter mortality of both Atlantic salmon and sea trout occur. Two other sites (Hjuksån and Ruskträskbäcken) are considered as reference sites that could potentially support anadromous fish since they have no migratory obstacle. However, no spawning grounds for Atlantic salmon or sea trout have been found here, nor has a high mortality of these species been found.

The streams were sampled in October 2014 (resident brown trout samples) and in the early spring (mid-April) 2015 [primary producers (periphyton) and benthic invertebrates], after the end of the ice cover and before the spring flood. The same kind of aquatic organisms (and the same size when possible) from all streams was collected. Periphyton were collected by taking five stones (10–15 cm in diameter) at each sampling site from different locations in the stream. The stones were placed into separate sample bags and then in a cooling dark box to avoid contamination with organic material during transportation to the laboratory. After this, the upper surface of these stones was immediately scrubbed with a toothbrush in the laboratory in order to remove the periphyton, which was then frozen for the later analyses.

Benthic invertebrates were collected by kicking the streambed upstream from a mesh net with the size of 200 μm. Collected invertebrates were emptied onto an enamel pan to sort out the organisms, which were then stored in a cooling box for transportation to the laboratory [7]. Benthic invertebrates were identified to the genus level when possible, otherwise to the family level. The selected invertebrates (1.5–2 cm total length, n = 3 per genus/family) were kept alive for 48 h in filtered (Whatman GF/F) stream water for gut evacuation [31,42]. The following benthic invertebrates were collected: mayflies (Ephemeroptera: *Baetis,* Heptageniidae, and *Ephemerella*), caddisflies (Trichoptera: *Hydropsyche*), and stoneflies (Plecoptera). Mayflies are mainly herbivore collectors/grazers, feeding on periphyton and detritus [43]. *Hydropsyche* is a filter-feeding insect with some grazing and predation [44]. Plecoptera are mostly herbivores/detritivores and generally found in cold, fast-moving streams [45]. Plecoptera were not identified to the genus level, but *Taeniopteryx nebulosa* (Taeniopterygidae), *Nemoura avicularis* Morton, and *Protonemura meyeri* (Pictet) (Nemouridae) are common in North European streams [46]. The samples were stored in separate labeled bags in the freezer until freeze-dried.

Electrofishing was used to collect brown trout from streams (n = 3 per stream). Total length of sampled brown trout ranged from 140 to 180 mm in Baggböleforsen compared to 125–160 mm in Djupseleforsen, 145–170 mm in Hjuksån, and 140–169 mm in Ruskträskbäcken. Sexually mature female Atlantic salmon and sea trout (n = 3 per species) were sampled during the autumn of 2013 at the Stornorrfors fish ladder when they were migrating upstream for spawning. Around ¼ of eggs were taken from each reproductive adult and frozen for further analyses.

### 2.2 Sample analyses

The number of samples analyzed for stable isotopes was three for each organism and site [31,42,47]. Dorsal muscle tissue samples were taken from each fish and were rinsed in a 10 % solution of HCl to remove inorganic carbonates and then in distilled water [7]. The studied organisms (periphyton, benthic invertebrates, and fish) and eggs were freeze-dried for 2 days [7,31]. The samples were then ground into a powder and stored in different glass vials. All glass vials were labeled and stored over silica gel desiccant [31]. Later, a specific amount of the dried samples (5 mg for periphyton and 0.5 mg for other organisms) from each glass vial was weighed and put into a tin capsule for the later SIA analysis. Nitrogen and carbon contents, as well as nitrogen (δ^15^N) and carbon (δ^13^C) stable isotopic composition, were analyzed using an Isotope Ratio Mass Spectrometer (DeltaV, Thermo Fisher Scientific, Bremen, Germany) interfaced with an Elemental Analyzer (EA-IRMS) (Flash EA 2000, Thermo Fisher Scientific, Bremen, Germany) at SLU's Stable Isotope Laboratory (Umeå, Sweden). Stable isotope ratios are calculated as deviations from standards following the formula:

(1) $$\delta^{15}N\;\text{or}\;\delta^{13}C=\left(\left(R_{\text{sample}}\;–R_{\text{standard}}\right)/R_{\text{standard}}\right)\times 1000$$

where R_sample_ and R_standard_ are the heavy-to-light isotope ratios (^15^N/^14^N and ^13^C/^12^C) of the samples and standards, respectively [48]. The international standard for nitrogen is atmospheric N_2_, and for carbon, it is a marine limestone called Peedee Belemnite [32,48]. C and N of the dried sample material is converted to CO_2_ and N_2_ by combustion, and working standards are wheat and maize flour calibrated against reference standards including IAEA-600, IAEA-N-2, USGS40, and USGS41 for nitrogen isotopes and IAEA-600, IAEA-CH-6, and USGS40 for carbon isotopes. δ^13^C values of aquatic animals were lipid-corrected according to the model for aquatic animals (Post et al., 2007).

### 2.3 Data analyses

A two-way ANOVA was used with fish species and egg/muscle as fixed factors separately for comparison of δ^15^N and δ^13^C values estimated in egg and tissue of reproductive adults. Generalized linear mixed models (GLMM) fitted by maximum likelihood estimation [49,50] were applied to analyze the effect of the presence of salmonid spawning grounds on nitrogen or carbon stable isotope ratios of three components of the food chain in the studied areas. “Site” (two levels (reference site/MDN site)) and “Food web component” (three levels (periphyton/benthic macroinvertebrates/brown trout) were used as categorical factors and “stream” as a random effect variable as follows:

(2) $$\delta^{15}N\;\text{or}\;\delta^{13}C=\beta_{0}+\beta_{1}\;\left(\text{Site}\right)+\beta_{2}\;\left(\text{Food}\;\text{web}\;\text{component}\right)+\beta_{3}\left(\mathrm{Site}\times \mathrm{Food}\;\mathrm{web}\;\mathrm{component}\right)+{\text{U}}_{\text{i}}\;\text{Stream}$$

where β0 is the model intercept and β1-β2 are specific coefficients of fixed variables and Ui is the random stream-specific intercept.

For comparison of δ^15^N and δ^13^C values among benthic macroinvertebrates between two sites, a nonparametric Kruskal–Wallis test was used, followed by a post hoc Dunn's test for multiple comparisons because the assumptions about homogeneity of variance were not met, as indicated by Levene's test of equality of error variances.

A Bayesian Mixing Model, implemented via the MixSIAR R package, was used to estimate the relative contributions of different benthic invertebrates (sources) to brown trout (consumer) tissues across the sampling sites [[51], [52], [53]]. Consumer species was specified as a fixed effect, and both residual and process errors were incorporated into the model's error structure. Two diagnostic tests, the Gelman-Rubin and Geweke diagnostics, were used to assess the Markov Chain Monte Carlo (MCMC) convergence. Values below 1.05 were considered indicative of good convergence [53]. If this threshold was not met, the analysis was repeated with a longer chain length [52,54]. The MCMC parameters in the JAGS model within MixSIAR were configured to include three chains with 30,000 iterations each, burn-in at 200,000, and thin at 100 [52]. The software used for running the model was R version 4.3.2. Statistical significance was accepted at the p < 0.05 level.

## 3 Results

### 3.1 Salmon and sea trout reproductive adults

**Fig. 2:**
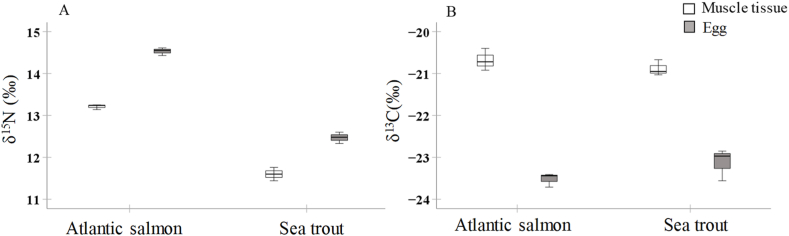
Stable nitrogen (A) and carbon (B) isotope ratios in Atlantic salmon and sea trout muscle and eggs.

Nitrogen and carbon isotope values for Atlantic salmon and sea trout tissue were higher than those for other food web components. ANOVA results revealed that δ^15^N and the δ^13^C values varied from 13.1 to 14.6 ‰ and −23.7 to −21.0 ‰ in anadromous Atlantic salmon ascending upstream compared to 11.4–12.6 ‰ and −23.6 to −21.3 ‰ in anadromous sea trout, respectively. Atlantic salmon had higher δ^15^N values than sea trout (F_1,9_ = 344.5, p < 0.001, Fig. 2A). Fish species had no significant effect on δ^13^C values of eggs/muscles (F_1,9_ = 0.02, p = 0.898, Fig. 2B). In both fish species, eggs were enriched in ^15^N (F_1,9_ = 122.7, p < 0.001, Fig. 2A) and depleted in ^13^C compared to muscle (F_1,9_ = 60.1, p < 0.001, Fig. 2B).

### 3.2 Food web components

**Fig. 3:**
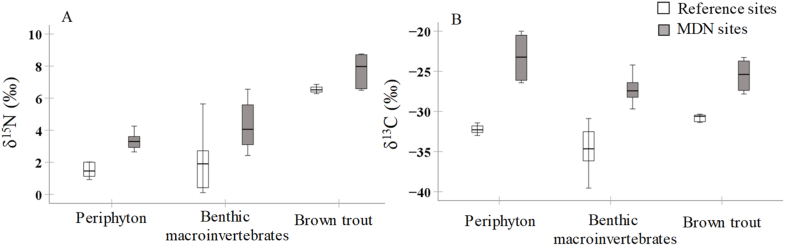
Stable nitrogen (A) and carbon (B) isotope ratios in food web components (periphyton, benthic macroinvertebrates, and brown trout) collected from MDN sites and references sites.

GLMM results revealed that nitrogen and carbon isotope values for individual food web components (n = 84) differed among sites (references and MDN sites) (Table 1). Response of organisms’ δ^15^N values to MDN was not different in various food web components as indicated by the non-significant interaction between Site and Food web component (Table 1, Fig. 3A). Nevertheless, a strong Site × Food web component interaction for the δ^13^C value indicated that the effects of site on δ^13^C of the studied organism differed among food web components (Table 1, Fig. 3B). All food web components, including periphyton, benthic invertebrates, and trout, had higher δ^15^N (Fig. 3A) and δ^13^C values (Fig. 3B) in MDN sites than in reference sites.

**Table 1:** Output of generalized linear mixed model testing the effects of “Site” (two levels (reference site/marine-derived nutrients site) and “Food web component” (three levels (periphyton/benthic macroinvertebrates/brown trout)) and their interaction on δ^15^N and δ^13^C values of the studied organisms.

		δ^15^N			δ^13^C	
Variable	χ^2^	DF	p-value	χ^2^	DF	p-value
Site	6.1	1	<0.05	38.1	1	<0.001
Food web component	143.7	2	<0.001	46.0	2	<0.001
Site × Food web component	2.9	2	0.2	6.4	2	<0.05

**Fig. 4:**
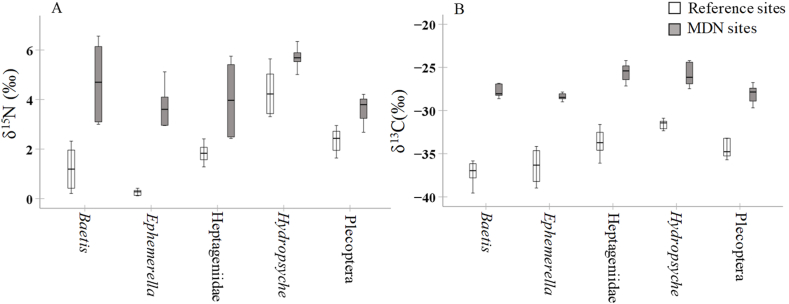
Stable nitrogen (A) and carbon (B) isotope ratios in different species/order of benthic macroinvertebrates collected from MDN sites and references sites.

Likewise, the Kruskal–Wallis test revealed that all species of benthic macroinvertebrates in MDN sites had higher δ^15^N and δ^13^C values than those in reference sites (p < 0.001, n = 60, Fig. 4AB). Species had a significant effect only on δ^15^N values (p < 0.01, Kruskal–Wallis test, n = 60, Fig. 4A) of benthic macroinvertebrates, while the differences in δ^13^C values among species were marginally significant (p = 0.068, Kruskal–Wallis test, n = 60, Fig. 4B). Among macroinvertebrates, regardless of sampling sites, *Hydropsyche* had the highest δ^15^N value (5 ‰) (Dunn's test, all p < 0.01, Fig. 4A). Although *Ephemerella* had the lowest δ^15^N value (1.99 ‰) among species, there were no significant differences in δ^15^N values of *Ephemerella*, *Baetis* (2.96 ‰), Heptageniidae (2.92 ‰), and Plecoptera (2.99 ‰) (Dunn's test, all p > 0.05, Fig. 4A). Regardless of sampling sites, the average δ^13^C values were −32.5 ‰ for *Baetis*, −29.6 ‰ for Heptageniidae, −28.7 ‰ for *Hydropsyche*, −32.4 ‰ for *Ephemerella*, and −31.3 ‰ for Plecoptera.

**Fig. 5:**
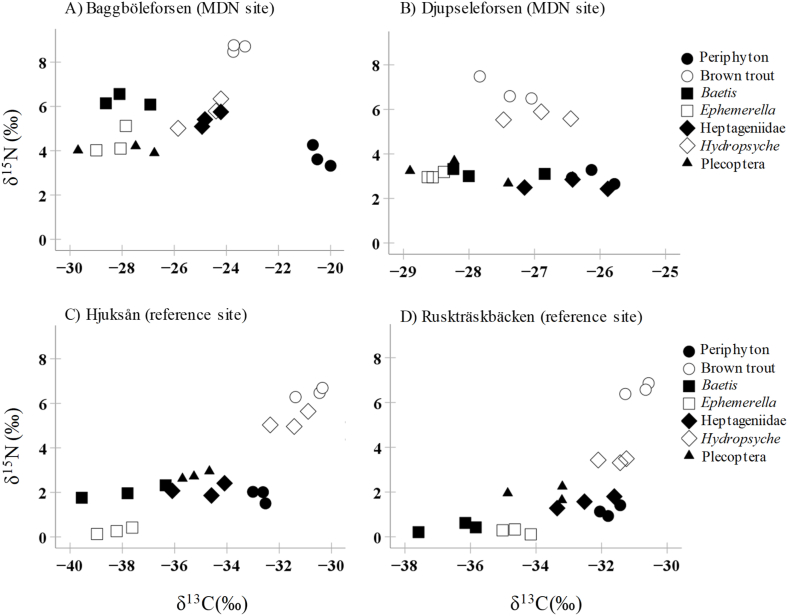
Distribution of carbon and nitrogen stable isotope ratios among samples collected from four streams. Circles enclose different species.

Distribution of carbon and nitrogen stable isotope ratios among samples collected from the streams revealed that different food web components were mostly differentiated in separate groups by their isotopic values (Fig. 5). In all streams, resident trout had the highest δ^15^N value, indicating that they are in higher trophic positions than other species (Fig. 5). Among primary consumers, *Hydropsyche* represented the highest δ^15^N value (Fig. 5). However, *Baetis* and *Hydropsyche* exhibited similar δ^15^N values in Baggböleforsen (Fig. 5). Generally, most of the food web components had higher δ^15^N values in Baggböleforsen (MDN site) than in other sites. *Ephemerella*'s trophic position was higher in both Baggböleforsen and Djupseleforsen (MDN sites) than in Hjuksån and Ruskträskbäcken (reference sites) (Fig. 5).

**Fig. 6:**
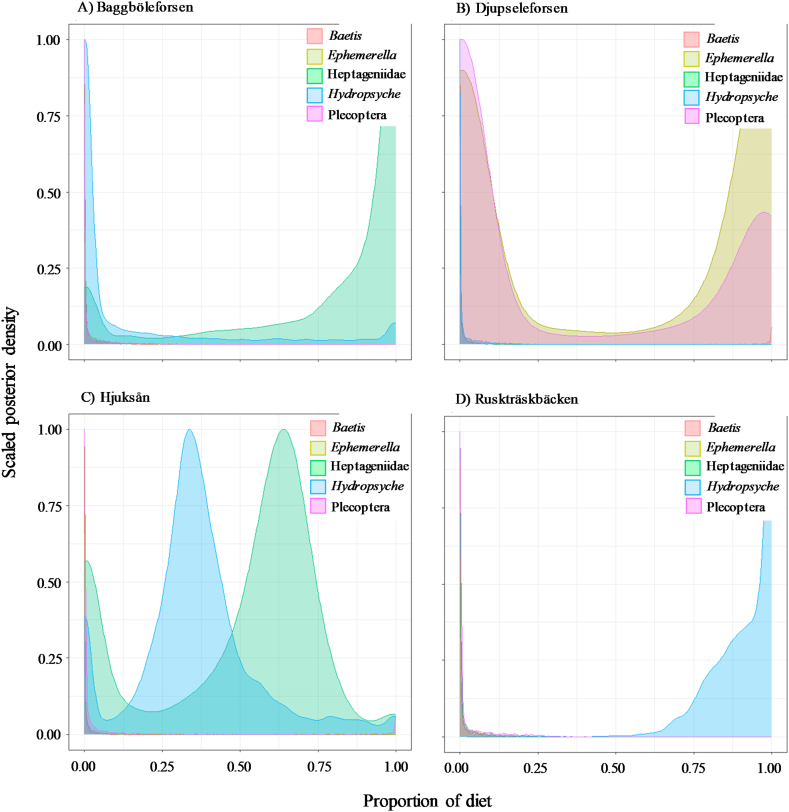
MixSIR results for the relative contributions of different benthic invertebrates (sources) to brown trout (consumer) tissues across our sampling sites.

The MixSIAR model showed variations in the diet of brown trout in different streams (Table 2, Fig. 6). The most important source of food for brown trout were Heptageniidae and Hydropsyche in Baggböleforsen, Ephemerella and Pleocoptera in Djupseleforsen, Heptageniidae, Hydropsyche, and Pleocoptera in Hjuksån and Hydropsyche in Ruskträskbäcken (Table 2, Fig. 6). It seems that only in Ruskträskbäcken trout fed mainly on one item (Hydropsyche) (Table 2, Fig. 6).

**Table 2:** Mean (±SD) values of food resource (benthic macroinvertebrates) contribution proportion to brown trout as determined by MixSIAR for our sampling sites.

Stream	Food resources	Mean ± SD
Baggböleforsen	*Baetis*	0.014 ± 0.044
	*Ephemerella*	0.019 ± 0.047
	Heptageniidae	0.787 ± 0.316
	*Hydropsyche*	0.155 ± 0.291
	Pleocoptera	0.025 ± 0.060
Djupseleforsen	*Baetis*	0.101 ± 0.274
	*Ephemerella*	0.533 ± 0.465
	Heptageniidae	0.010 ± 0.038
	*Hydropsyche*	0.018 ± 0.042
	Pleocoptera	0.338 ± 0.440
Hjuksån	*Baetis*	0.029 ± 0.106
	*Ephemerella*	0.024 ± 0.071
	Heptageniidae	0.482 ± 0.277
	*Hydropsyche*	0.363 ± 0.192
	Pleocoptera	0.102 ± 0.266
Ruskträskbäcken	*Baetis*	0.014 ± 0.032
	*Ephemerella*	0.015 ± 0.036
	Heptageniidae	0.026 ± 0.070
	*Hydropsyche*	0.912 ± 0.102
	Pleocoptera	0.033 ± 0.071

## 4 Discussion

The results from this study demonstrate that all food web components studied are enriched with ^15^N and ^13^C in MDN sites (compared to the reference sites) where Atlantic salmon and sea trout spawn and the overwinter mortality occurs. This indicates a potential contribution of Atlantic salmon and sea trout to the nutrient dynamics of these boreal streams. This study also lends support to previous studies showing that anadromous iteroparous salmonids could be important vectors for nutrient fluxes between marine and freshwater ecosystems [7,20,22,23].

High values of δ^15^N and δ^13^C in anadromous salmonids in the current study indicate that they sequestered high amounts of MDN in their bodies. However, it is also possible that fractionation among isotopes occurs from resources other than MDN. These nitrogen and carbon isotope values are comparable with the findings from other studies on Atlantic salmon in the Baltic Sea. For example, Berglund et al. (2001) reported stable isotope values of 11.7–13.7 ‰ for δ^15^N and −22.0 to −19.6 ‰ for δ^13^C in the entire population of Atlantic salmon [38]. Similarly, Persson et al. (2007) found an average δ^15^N value of 14.4 ‰ and an average δ^13^C value of −21.55 ‰ for Atlantic salmon (Fork length = 79 cm, n = 29) in the southern Baltic Sea [39]. However, by using stable isotope analysis of archived scales (1989–2011) of Atlantic salmon (73–90 cm) collected from five areas (River Simojoki, River Kymijoki, Baltic Proper, Bothnian Sea, Gulf of Finland), Torniainen et al. (2014) reported stable isotope values of 11.2–14.5 ‰ for δ^15^N and −15.5 to −18.0 ‰ for δ^13^C [40]. Similarly, synthesis of N and C data for the five species of Pacific salmon revealed δ^15^N values of 10.5–15.2 ‰ and δ^13^C values of −22.5–17.8 ‰ ([37] and references therein). Furthermore, Atlantic salmon eggs had higher δ^15^N values and lower δ^13^C values than the muscles of the spawning fish, corresponding well to what Bilby et al. (1996) found in Pacific salmon. The ^13^C difference in muscle versus egg can also partially be due to their different lipid contents, given that lipids are more depleted in ^13^C and eggs contain more lipids [55].

**Fig. 7:**
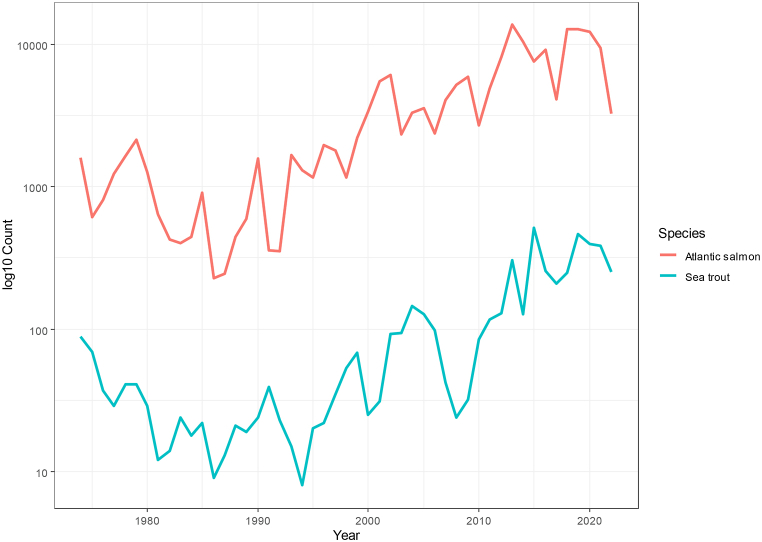
Numbers of anadromous wild salmon spawners in fishways and hydroacoustic counting in River Ume/Vindelälven (A) and number of anadromous (wild and stocked) sea trout spawners from fish counters in River Vindelälven (B) during 1974–2022.

Long-term data shows an increasing number of anadromous wild Atlantic salmon spawners in River Ume/Vindelälven (Fig. 7) and (wild + stocked) sea trout spawners in River Vindelälven (Fig. 7) from 1974 to 2022 [56] [Katarina Magnusson pers. comm.]. These anadromous salmonids can potentially transport a large amount of MDN to these freshwater ecosystems through overwinter mortality and production of eggs/sperms. During 2015–2018, however, the number of anadromous Atlantic salmon spawners decreased in Ume/Vindelälven owing to reduction in the passing success of salmon spawners through the fish ladder (Fig. 7) [56]. Nevertheless, the Atlantic salmon spawning run into the river has improved recently during 2019–2021 (Fig. 7) [56]. It appears that most of the MDN in the MDN sites are likely transported by Atlantic salmon due to their higher abundance during upstream migration compared to sea trout (Fig. 7) and because survival rates of post-spawners in Swedish streams are shown to be 40 % for Atlantic salmon and 95 % for sea trout [57].

Nearly all trophic levels (from periphyton to fish) from the MDN sites exhibited higher values of δ^15^N and δ^13^C than those from the reference sites. In streams affected by MDN of Pacific salmon, nutrients are not only slowly released from the decomposing carcasses and eggs [58] but also derive from the excretion by live Pacific salmon spawners [59]. These nutrients are then incorporated into the stream's food web following their uptake by primary producers and trophic transfer, or through direct consumption of carcasses and eggs by benthic macroinvertebrates and fishes [2,5,7,60,61]. Several studies have also indicated that Atlantic salmon MDN play a critical role for freshwater ecosystems [11,13,[20], [21], [22], [23],^25^,^26^]. MDN from excretion and gametes produced by spawning Atlantic salmon are predicted to enhance freshwater productivity, helping to alleviate the ‘bottom-up’ constraints currently affecting nutrient-limited systems [26]. It is also demonstrated that a relatively modest addition of simulated carcasses of Atlantic salmon could strongly boost the productivity of the freshwater food webs [13] through incorporation of MDN at multiple trophic levels [13,36,62]. After Atlantic salmon spawning in each year, Bryson et al. (2021) observed increased δ^13^C and δ^15^N values in all organisms at the downstream site of the Upper Salmon River (Canada), which was stocked by Atlantic salmon [63]. Further, Näslund et al. (2015) demonstrated that juvenile Atlantic salmon and brown trout consume eggs from anadromous salmonids in a Swedish coastal stream. They suggested that the nutritious eggs may positively affect their subsequent smoltification and winter survival rate [24]. It is likely that ^15^N enrichment in brown trout in MDN sites can partly be explained by their consumption of eggs with high δ^15^N value. However, isotopically elevated baseline of the entire system could also lead to the highest values of δ ^15^N and δ ^13^C of the organisms in streams.

*Hydropsyche* and *Ephemerella* had the highest and the lowest values of δ^15^N among benthic invertebrates, respectively. Nitrogen stable isotopic composition of food sources can be reflected in isotopic values of the consumers. In general, Hydropsychids filter organic matter from the water column [64]. The species of *Hydropsyche* were not identified in this study. However, in a laboratory experiment, Englund (1992) found that *Hydropsyche siltalai* is an important predator of *Ephemerella ignita* nymphs and *Simulium truncatum* larvae. Given the enrichment in ^15^N at each trophic level [32], *Hydropsyche* might occupy the highest trophic position among collected benthic macroinvertebrates. This species, according to MixSIAR, is one of the dominant food sources in the diet of brown trout in all streams except Djupseleforsen, leading to a high value of δ^15^N in this consumer. Compared to other macroinvertebrates, the lower isotopic values of *Ephemerella* may be explained by its feeding on a diet with even lower δ^15^N and δ^13^C values compared to periphyton.

A caveat of the current study is the independence of sites used in the study in terms of their exposure to the MDN from the spawning and dying of anadromous fishes. River and stream networks have an intrinsic tail-up dependency between downstream locations, such that a site downstream of a spawning site could receive MDN from that event, especially compared to sites upstream of it. In this case, the effect could have weakened the differences in *δ*^15^N and *δ*^13^C between the MDN and reference sites. There are also some caveats associated with the use of stable isotopes. For instance, the application of this technique relies on assumptions that are not well understood and have rarely been tested, and thus the collection of filed data should be complemented by laboratory experiments to validate the relevant assumptions [65]. Moreover, the inferences derived from one-compartment models to describe the dynamics of isotopic incorporation into animal tissues can differ from those derived from recent multi-compartment models [66]. Furthermore, tissue to diet discrimination factors, which are required in the mixing model, are rarely measured experimentally, and error in these factors can lead to incorrect estimates of source proportions [66]. The assumption used in mixing models that assimilated nutrients are broken down into their elemental components, which are then reassembled into biomolecules, is also unrealistic [66]. Further, differences in isotopic incorporation rates among tissues can be attributed to intertissue variations in protein turnover [66].

Since the turnover rates of isotopes are correlated with body mass [34], it was important to find a proper time for the sampling. In this study, trout samples were not collected at the same time as other food web samples. When it comes to benthic invertebrates, which have shorter turnover rates, it is critical to select the optimal time and also to sample all the streams within a short period of time to get an accurate result [34]. However, when it comes to large organisms like fish, the isotopic signature is representative of their diet over long periods of time [34], indicating that the sampling time is probably less important. In the current study, benthic macroinvertebrates were small and short-lived, and their isotope values can vary monthly. Brown trout, on the other hand, are longer-lived, with longer tissue turnover times. Thus, while benthic macroinvertebrates are reflecting short-term variations in food source, the trout muscle isotope values are integrated over longer time periods. Still, trout from the MDN sites exhibited the highest values of δ^15^N and δ^13^C in the current study. Furthermore, benthic macroinvertebrates were sampled in the early spring, after the end of ice cover and over-winter mortality, when salmon carcasses had already been decomposed. It seems that this is the best time to detect MDN in the studied stream biota, given that Bilby et al. (1996) found the highest levels of enrichment of the stream communities in the early spring [7]. However, MDN may stay in the food web for a longer time post-spawning, which can in turn lead to a gradual accumulation of MDNs in the aquatic ecosystem [63].

## 5 Conclusion

In conclusion, this study showed that stream food web components have different isotopic signatures depending on whether iteroparous salmonid spawning/mortality occurs at the test site or not. High δ^15^N and δ^13^C values in all food web components studied in the MDN site can be indications of a transport of MDN by the upstream migration of anadromous salmonids in the Ume/Vindel river system. These nutrients, which are most likely derived from fish carcasses and eggs as well as from the excretion of live anadromous fish spawners, are incorporated into the organisms of the streams where the salmonids breed and die and are likely important for nutrient dynamics and sustainability of these areas. Since both Atlantic salmon and sea trout are experiencing declining stocks, the information gained from this study could improve the understanding of ecosystem consequences of these declines. Therefore, it could serve as a first step warranting future studies that more explicitly aim to quantify the role of MDN in subsidizing these systems.

## CRediT authorship contribution statement

**Rahmat Naddafi:** Writing – original draft, Supervision, Software, Investigation, Funding acquisition, Formal analysis, Data curation, Conceptualization. **Magnus Enbom:** Methodology, Investigation, Data curation. **Carin Magnhagen:** Writing – original draft, Visualization, Validation, Methodology. **Nazila Hajizadeh Koupayh:** Writing – review & editing, Investigation, Data curation. **Daniel Holmqvist:** Writing – review & editing, Methodology, Investigation, Conceptualization. **Hans Lundqvist:** Writing – review & editing, Resources, Project administration.

## Ethics statement

Swedish guidelines were followed concerning the care and welfare of all collected fish. The methodology of this study was approved by the Swedish University of Agricultural Sciences Ethical Committee (License no. A58/12).

## Data availability statement

Data will be made available on request.

## Declaration of competing interest

The authors declare that they have no known competing financial interests or personal relationships that could have appeared to influence the work reported in this paper.

## References

[1] Polis G.A., Anderson W.B., Holt R.D. (1997). Toward an integration of landscape and food web ecology: the dynamics of spatially subsidized food webs. Annu. Rev. Ecol. Systemat..

[2] Vanni M.J. (2002). Nutrient cycling by animals in freshwater ecosystems. Annu. Rev. Ecol. Systemat..

[3] Gende S.M., Edwards R.T., Willson M.F., Wipfli M.S. (2002). Pacific salmon in aquatic and terrestrial ecosystems: Pacific salmon subsidize freshwater and terrestrial ecosystems through several pathways, which generates unique management and conservation issues but also provides valuable research opportunities. Bioscience.

[4] Janetski D.J., Chaloner D.T., Tiegs S.D., Lamberti G.A. (2009). Pacific salmon effects on stream ecosystems: a quantitative synthesis. Oecologia.

[5] Naiman R.J., Bilby R.E., Schindler D.E., Helfield J.M. (2002). Pacific salmon, nutrients, and the dynamics of freshwater and riparian ecosystems. Ecosystems.

[6] Walsh J.C., Pendray J.E., Godwin S.C., Artelle K.A., Kindsvater H.K., Field R.D., Harding J.N., Swain N.R., Reynolds J.D. (2020). Relationships between Pacific salmon and aquatic and terrestrial ecosystems: implications for ecosystem‐based management. Ecology.

[7] Bilby R.E., Fransen B.R., Bisson P.A. (1996). Incorporation of nitrogen and carbon from spawning coho salmon into the trophic system of small streams: evidence from stable isotopes. Can. J. Fish. Aquat. Sci..

[8] Hocking M.D., Reynolds J.D. (2011). Impacts of salmon on riparian plant diversity. Science.

[9] Childress E.S., Allan J.D., McIntyre P.B. (2014). Nutrient subsidies from iteroparous fish migrations can enhance stream productivity. Ecosystems.

[10] Claeson S.M., Li J.L., Compton J.E., Bisson P.A. (2006). Response of nutrients, biofilm, and benthic insects to salmon carcass addition. Can. J. Fish. Aquat. Sci..

[11] Guyette M.Q., Loftin C.S., Zydlewski J. (2013). Carcass analog addition enhances juvenile Atlantic salmon (Salmo salar) growth and condition. Can. J. Fish. Aquat. Sci..

[12] Kline T.C., Goering J.J., Piorkowski R.J. (1997). The effect of salmon carcasses on Alaskan freshwaters. Freshwaters of Alaska, Springer.

[13] McLennan D., Auer S.K., Anderson G.J., Reid T.C., Bassar R.D., Stewart D.C., Cauwelier E., Sampayo J., McKelvey S., Nislow K.H. (2019). Simulating nutrient release from parental carcasses increases the growth, biomass and genetic diversity of juvenile Atlantic salmon. J. Appl. Ecol..

[14] Muñoz N.J., Reid B., Correa C., Neff B.D., Reynolds J.D. (2020). Non‐native Chinook salmon add nutrient subsidies and functional novelty to Patagonian streams. Freshw. Biol..

[15] Nislow K.H., Armstrong J.D., McKelvey S. (2004). Phosphorus flux due to Atlantic salmon (Salmo salar) in an oligotrophic upland stream: effects of management and demography. Can. J. Fish. Aquat. Sci..

[16] Williams K., Griffiths S.W., Nislow K., McKelvey S., Armstrong J. (2009). Response of juvenile Atlantic salmon, Salmo salar, to the introduction of salmon carcasses in upland streams. Fish. Manag. Ecol..

[17] Jardine T.D., Roussel J.-M., Mitchell S.C., Cunjak R.A. (2009).

[18] Jonsson N., Hansen L.P., Jonsson B. (1991). Variation in age, size and repeat spawning of adult Atlantic salmon in relation to river discharge. J. Anim. Ecol..

[19] Klemetsen A., Amundsen P.A., Dempson J., Jonsson B., Jonsson N., O'connell M., Mortensen E. (2003). Atlantic salmon Salmo salar L., brown trout Salmo trutta L. and Arctic charr Salvelinus alpinus (L.): a review of aspects of their life histories. Ecol. Freshw. Fish.

[20] Elliott J., Lyle A., Campbell R. (1997). A preliminary evaluation of migratory salmonids as vectors of organic carbon between marine and freshwater environments. Sci. Total Environ..

[21] Guyette M.Q., Loftin C.S., Zydlewski J., Cunjak R. (2014). Carcass analogues provide marine subsidies for macroinvertebrates and juvenile A tlantic salmon in temperate oligotrophic streams. Freshw. Biol..

[22] Jonsson B., Jonsson N. (2003). Migratory Atlantic salmon as vectors for the transfer of energy and nutrients between freshwater and marine environments. Freshw. Biol..

[23] Lyle A., Elliott J. (1998). Migratory salmonids as vectors of carbon, nitrogen and phosphorus between marine and freshwater environments in north-east England. Sci. Total Environ..

[24] Näslund J., Aldvén D., Závorka L. (2015). Eggs from anadromous adults provide marine-derived nutrients to Atlantic salmon and brown trout parr in late autumn–observations from a Swedish coastal stream. Environ. Biol. Fish..

[25] Samways K.M., Blair T.J., Charest M.A., Cunjak R.A. (2017). Effects of spawning Atlantic salmon (Salmo salar) on total lipid content and fatty acid composition of river food webs. Ecosphere.

[26] Samways K.M., Cunjak R.A. (2015). Increases in benthic community production and metabolism in response to marine‐derived nutrients from spawning A tlantic salmon (S almo salar). Freshw. Biol..

[27] Samways K.M., Quiñones-Rivera Z.J., Leavitt P.R., Cunjak R.A. (2015). Spatiotemporal responses of algal, fungal, and bacterial biofilm communities in Atlantic rivers receiving marine-derived nutrient inputs. Freshw. Sci..

[28] Finenko G.A., Kideys A.E., Anninsky B.E., Shiganova T.A., Roohi A., Tabari M.R., Rostami H., Bagheri S. (2006). Invasive ctenophore Mnemiopsis leidyi in the Caspian Sea: feeding, respiration, reproduction and predatory impact on the zooplankton community. Mar. Ecol. Prog. Ser..

[29] Ghodrati F., Ghorbani R., Agh N., Hedayati A., Naddafi R., Jalali A., Shiroudmirzaei F. (2022). Assessing nitrogen dynamics model and the role of artificial lagoon in effluent loading of shrimp farms in Gomishan wetland, southern Caspian Sea. Sci. Rep..

[30] Ghojoghi A., Ghorbani R., Patimar R., Salmanmahiny A., Naddafi R., Fazel A., Jardine T.D. (2023). The fate of nitrogen in the Zarin-Gol River receiving trout farm effluent. Sci. Rep..

[31] Naddafi R., Koupayeh N.H., Ghorbani R. (2021). Spatial and temporal variations in stable isotope values (δ13C and δ15N) of the primary and secondary consumers along the southern coastline of the Caspian Sea. Mar. Pollut. Bull..

[32] Peterson B.J., Fry B. (1987). Stable isotopes in ecosystem studies. Annu. Rev. Ecol. Systemat..

[33] Vander Zanden H.B., Soto D.X., Bowen G.J., Hobson K.A. (2016). Expanding the isotopic toolbox: applications of hydrogen and oxygen stable isotope ratios to food web studies. Front. Ecol. Evol..

[34] Post D.M. (2002). Using stable isotopes to estimate trophic position: models, methods, and assumptions. Ecology.

[35] McCutchan J.H., Lewis W.M., Kendall C., McGrath C.C. (2003). Variation in trophic shift for stable isotope ratios of carbon, nitrogen, and sulfur. Oikos.

[36] Samways K., Soto D., Cunjak R. (2018). Aquatic food‐web dynamics following incorporation of nutrients derived from Atlantic anadromous fishes. J. Fish. Biol..

[37] Johnson S.P., Schindler D.E. (2009). Trophic ecology of Pacific salmon (Oncorhynchus spp.) in the ocean: a synthesis of stable isotope research. Ecol. Res..

[38] Berglund O., Larsson P., Broman D. (2001). Organochlorine accumulation and stable isotope ratios in an Atlantic salmon (Salmo salar) population from the Baltic Sea. Sci. Total Environ..

[39] Persson M., Larsson P., Stenroth P. (2007). Fractionation of δ15N and δ13C for Atlantic salmon and its intestinal cestode Eubothrium crassum. J. Fish. Biol..

[40] Torniainen J., Vuorinen P.J., Jones R.I., Keinänen M., Palm S., Vuori K.A., Kiljunen M. (2014). Migratory connectivity of two Baltic Sea salmon populations: retrospective analysis using stable isotopes of scales. ICES (Int. Counc. Explor. Sea) J. Mar. Sci..

[41] Lundqvist H., Rivinoja P., Leonardsson K., McKinnell S. (2008). Proceedings of the Symposium Held 29 March–1 April 2005, Bordeaux, France.

[42] Premke K., Karlsson J., Steger K., Gudasz C., von Wachenfeldt E., Tranvik L.J. (2010). Stable isotope analysis of benthic fauna and their food sources in boreal lakes. J. North Am. Benthol. Soc..

[43] Brittain J.E. (1982). Biology of mayflies. Annu. Rev. Entomol..

[44] Englund G. (1992).

[45] Stewart K.W. (2009).

[46] Jonsson M., Malmqvist B. (2000). Ecosystem process rate increases with animal species richness: evidence from leaf‐eating, aquatic insects. Oikos.

[47] Friberg N., Dybkjaer J.B., Olafsson J.S., Gislason G.M., Larsen S.E., Lauridsen T.L. (2009). Relationships between structure and function in streams contrasting in temperature. Freshw. Biol..

[48] Fry B. (2006).

[49] Littell R.C., Milliken G.A. (2006).

[50] Naddafi R., Östman Ö., Bergström L., Mustamäki N., Appelberg M., Olsson J. (2022). Improving assessments of coastal ecosystems–Adjusting coastal fish indicators to variation in ambient environmental factors. Ecol. Indicat..

[51] Parnell A.C., Phillips D.L., Bearhop S., Semmens B.X., Ward E.J., Moore J.W., Jackson A.L., Grey J., Kelly D.J., Inger R. (2013). Bayesian stable isotope mixing models. Environmetrics.

[52] Stock B.C., Jackson A.L., Ward E.J., Parnell A.C., Phillips D.L., Semmens B.X. (2018). Analyzing mixing systems using a new generation of Bayesian tracer mixing models. PeerJ.

[53] Stock B.C., Semmens B.X. (2016).

[54] McCauley D.J., Young H.S., Dunbar R.B., Estes J.A., Semmens B.X., Micheli F. (2012). Assessing the effects of large mobile predators on ecosystem connectivity. Ecol. Appl..

[55] Arostegui M.C., Schindler D.E., Holtgrieve G.W. (2019). Does lipid-correction introduce biases into isotopic mixing models? Implications for diet reconstruction studies. Oecologia.

[56] Du Ciem S. (2021).

[57] Lundqvist H., Leonardsson K., Williams J., Östergren J., Hellström G., Forssén Å. (2015).

[58] Parmenter R.R., Lamarra V.A. (1991). Nutrient cycling in a freshwater marsh: the decomposition of fish and waterfowl carrion. Limnol. Oceanogr..

[59] Tiegs S.D., Levi P.S., Rüegg J., Chaloner D.T., Tank J.L., Lamberti G.A. (2011). Ecological effects of live salmon exceed those of carcasses during an annual spawning migration. Ecosystems.

[60] Kaylor M.J., White S.M., Sedell E.R., Sanders A.M., Warren D.R. (2020). Carcass additions influence food webs through bottom-up and direct consumption pathways along a fish species assemblage gradient. Ecosystems.

[61] Richardson D.P., Kohler A.E., Hailemichael M., Finney B.P. (2017). The fate of marine-derived nutrients: tracing δ13C and δ15N through oligotrophic freshwater and linked riparian ecosystems following salmon carcass analog additions. Can. J. Fish. Aquat. Sci..

[62] Nislow K., Kennedy B., Armstrong J., Collen P., Keay J., McKelvey S. (2010). Salmonid Fisheries: Freshwater Habitat Management.

[63] Bryson G.E., Kidd K.A., Samways K.M. (2022). Food web incorporation of marine-derived nutrients after the reintroduction of endangered inner Bay of Fundy Atlantic salmon (Salmo salar). Can. J. Fish. Aquat. Sci..

[64] Voelz N.J., Ward J. (1992). Feeding habits and food resources of filter-feeding Trichoptera in a regulated mountain stream. Hydrobiologia.

[65] Gannes L.Z., O'Brien D.M., Del Rio C.M. (1997). Stable isotopes in animal ecology: assumptions, caveats, and a call for more laboratory experiments. Ecology.

[66] Martínez del Rio C., Wolf N., Carleton S.A., Gannes L.Z. (2009). Isotopic ecology ten years after a call for more laboratory experiments. Biol. Rev..

