# Supplementary material for: The presence of iteroparous salmonid spawning grounds affects the stable isotope signatures of food web components in Swedish boreal streams
Source: Heliyon. 2025 Jan 23;11(3):e42173. doi: 10.1016/j.heliyon.2025.e42173 (PMC11800077; doi:10.1016/j.heliyon.2025.e42173)
Supplement: Multimedia component 1 [file mmc1.docx]

**Supplementary material**

**Study area**

*Description of sites*

1) Djupseleforsen (sampling site: 65°11'44.6"N, 18°13'15.9"E): mean highest discharge (MHQ); 843.765, Mean discharge (MQ);146.362, mean low flow discharge (MLQ): 23.669 (<https://vattenweb.smhi.se/hydronu/>). It is located approximately 180km upstream Stornorrfors. Main channel (Vindelälven) was dredged/channelized due to timber floating at sampling site but have suitable nursing habitats and spawning habitats nearby. According to electrofishing data, Djupseleforsen with high densities of salmon and moderate densities of brown trout is one of the largest spawning sites for salmon in the catchment area of the Vindelälven.

2) Baggböleforsen (sampling site: 63°50'14.0"N 20°7'24.6"E): Baggböleforsen is a stream with high winter mortality of salmon and sea trout downstream from the hydropower station in Stornorrfors. In fact, it is the old river bed, from the confluence area to the fish ladder downstream of the dam in Norrfors (a village in Umeå municipality), which used to serve as migration route during summer. This stream carries water through the dam only between 20^th^ May and 30^th^ September (<https://group.vattenfall.com/se/siteassets/sverige/var-verksamhet/energislag/tappningsschema-stornorrfors-2024.pdf>). When water flow rate is dropped to 0.8 m^3^/s from 1^st^ October, spawning parents are trapped below the dam and higher mortality rate is observed in Baggböleforsen compared to unregulated streams.

3) Hjuksån (sampling site: 64°22'1.4"N 19°36'7.3"E): MHQ: 21.457, MQ: 3.98, MLQ: 0.847 (<https://vattenweb.smhi.se/hydronu/>). It is a medium sized tributary, located approximately 55km upstream Stornorrfors and was dredged/channelized due to timber floating. This stream was restored in 2010 following the Vindelälvenr LIFE project where nursing areas were improved by adjusting stones and boulders and spawning areas by adding external gravel.

4) Ruskträskbäcken (sampling site: 64°49'33.3"N 18°45'39.3"E): MHQ: 11.009, MQ: 3.133, MLQ: 1.014 (<https://vattenweb.smhi.se/hydronu/>). It is a medium sized tributary, located approximately 125km upstream Stornorrfors and was dredged/channelized due to timber floating. This stream was restored in 2005 and 2010 following the Vindelälven LIFE project where nursing areas were improved by adjusting stones and boulders and spawning areas by adding external gravel.

*Benthic macroinvertebrates*

Data for benthic macroinvertebrates in Hjuksån and Ruskträskbäcken are lacking. Instead, we present benthic macroinvertebrates data for the closest tributaries. Storkvarnbäcken is in the same catchment area as Hjuksån, ca 5km upstream site. In an EVP-project (2002-2005), among 37 species (7762 individuals) found in Storkvarnbäcken and among 36 species (4145 individuals) found in Målten, mayflies, stoneflies, and caddisflies are almost the dominant benthic invertebrates (Table S1). There is no benthic invertebrate data in Baggböle and Djupseleforsen but we expect that benthic invertebrates in these two sites are similar to the aforementioned tributaries since Storkvarnbäcken and Maltån are tributaries to Vindelälven, which is tributary to Umeälven. This also explains why we were able to find similar benthic invertebrates in our four sampling streams, which were also confirmed by our visual observations.

*Physicochemical data*

Physicochemical data was provided only for the Hjuksån and Ruskträskbäcken (Table S2). There was no data physicochemical data available for other sites.

Table S1- Dominant benthic macroinvertebrates in Storkvarnbäcken and Målten during 2002-2005. Storkvarnbäcken is in the same catchment area as Hjuksån ca 5km upstream site. Maltån is the closest stream to Ruskträskbäcken, with approximately 30 km distance from sampling site.

| **Storkvarnbäcken** |  | **Målten** |  |
| --- | --- | --- | --- |
| **Species** | **Percentage** | **Species** | **Percentage** |
| *Baetis rhodani* | 26,11 | *Baetis rhodani* | 46,15 |
| *Amphinemura sulcicollis* | 21,09 | *Amphinemura borealis* | 12,86 |
| *Leuctra hippopus* | 8,57 | *Baetis niger* | 10,54 |
| *Baetis muticus* | 7,79 | *Protonemura meyeri* | 9,12 |
| *Protonemura meyeri* | 6,92 | *Simuliidae* | 3,59 |
| *Amphinemura borealis* | 6,75 | *Heptagenia sulphurea* | 3,38 |
| *Baetis niger* | 6,51 | *Leuctra hippopus* | 2,41 |
| *Isoperla grammatica* | 2,99 | *Capnopsis schilleri* | 1,83 |
| *Heptagenia sulphurea* | 2,69 | *Amphinemura sulcicollis* | 1,81 |
| *Hydropsyche pellucidula* | 1,33 | *Oligochaeta* | 1,23 |
| *Oligochaeta* | 1,30 | *Ephemerella mucronata* | 1,09 |
| *Leptophlebia vespertina* | 1,21 | *Taeniopteryx nebulosa* | 1,06 |
| *Chironomidae* | 1,06 |  |  |
| *Simuliidae* | 1,00 |  |  |

| **Parameter** | **Stream** | **2014-12-10** | **2015-03-15** | **2015-04-15** | **2015-05-05** | **2015-05-12** | **2015-05-19** | **2015-05-25** | **2015-06-02** | **2015-07-02** | **2015-08-10** | **2015-09-10** |
| --- | --- | --- | --- | --- | --- | --- | --- | --- | --- | --- | --- | --- |
| DOC | Ruskträskbäcken | 6.01 | 6.00 | 6.27 | 4.74 | 5.22 | 5.28 | 5.16 | 4.99 | 5.31 | 5.29 | 5.70 |
|  | Hjuksån | 13.30 | 12.20 | 16.22 | 12.88 | 12.73 | 12.05 | 12.03 | 11.72 | 11.05 | 10.82 | 10.30 |
| pH | Ruskträskbäcken | 6.75 | 6.8 | 6.6 | 6.65 | 6.66 | 6.7 | 7.13 | 6.76 | 6.76 | 7.04 | 7.1 |
|  | Hjuksån | 6.35 | 6.52 | 6.13 | 6.01 | 6.1 | 6.17 | 6.36 | 6.21 | 6.36 | 6.37 | 6.49 |
| Ntot | Ruskträskbäcken | 0.23 | 0.13 | 0.28 | 0.12 | 0.16 | 0.18 | 0.16 | 0.14 | 0.19 | 0.16 | 0.16 |
|  | Hjuksån | 0.35 | 0.22 | 0.36 | 0.20 | 0.26 | 0.25 | 0.26 | 0.25 | 0.28 | 0.28 | 0.28 |
| NO3 | Ruskträskbäcken | 10.00 | 22.75 | 16.09 | 13.79 | 11.98 | 6.61 | 4.43 | 4.07 | 3.4 | 3.18 | - |
|  | Hjuksån | 18.71 | 43.74 | 17.8 | 11.62 | 7.14 | 8.04 | 6.47 | 4.75 | 5.37 | 4.52 | - |
| NH4 | Ruskträskbäcken | 20.72 | 38.6 | 12.31 | 4.71 | 6.07 | 1.59 | 2.18 | 4.13 | 2.53 | 2.18 | - |
|  | Hjuksån | 21.3 | 10.58 | 5.35 | 5.22 | 5.8 | 7.32 | 4.33 | 3.69 | 6.44 | 2.37 | - |
| PO4 | Ruskträskbäcken | 1.19 | 0.69 | 0.5 | 0.87 | 1.4 | 0.03 | 0.25 | 0.02 | 0.13 | 0,00 | - |
|  | Hjuksån | 1.37 | 1.85 | 0.85 | 1.2 | 1.28 | 0.11 | 0.09 | 0.2 | 0.6 | 0.17 | - |

Table S2. Physicochemical parameters of the reference streams during December 2014-September 2015. Data for Ruskträskbäcken was not available and the data for Falåströmsbäcken, a site in the same watershed as Ruskträskbäcken was used.
